# Supplementary material for: Ligand recognition and biased agonism of the D1 dopamine receptor
Source: Nat Commun. 2022 Jun 8;13:3186. doi: 10.1038/s41467-022-30929-w (PMC9177848; doi:10.1038/s41467-022-30929-w)
Supplement: Supplementary file 1 — Supplementary Information [file 41467_2022_30929_MOESM1_ESM.pdf]

SUPPLEMENTARY INFORMATION

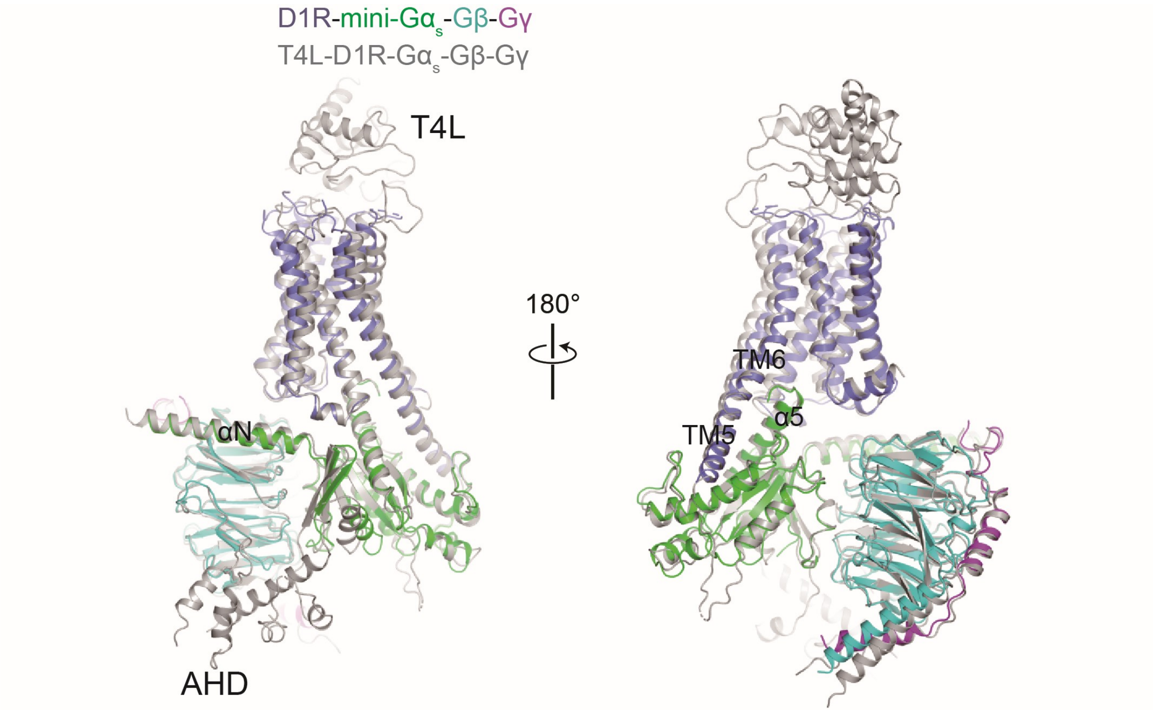

Supplementary Fig. 1. Structural overlay of the cryo-EM structure of D1R-minGs complex and the crystal structure of this complex (PDB ID: 7JOZ) in two opposite views.

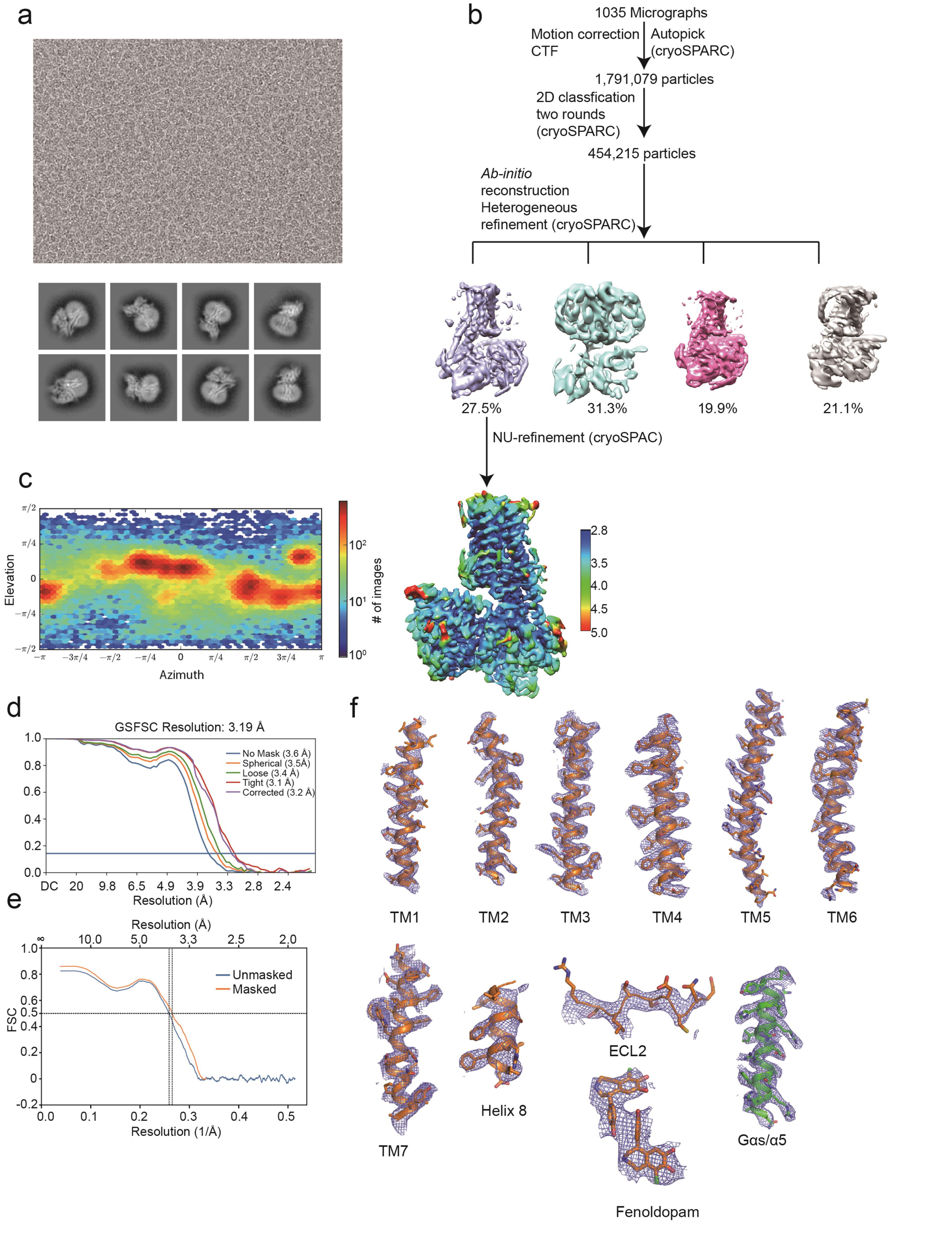

Supplementary Fig. 2. Cryo-EM data processing for the fenoldopam-bound D1R complex. a, Representative cryo-EM image (top) and 2D class average (bottom). **b,** Cryo-EM workflow chart. **c,** Angular distribution plot. **d,** Gold standard FSC curves. **e,** FSC curves of model-to-map. **f,** Representative EM density map.

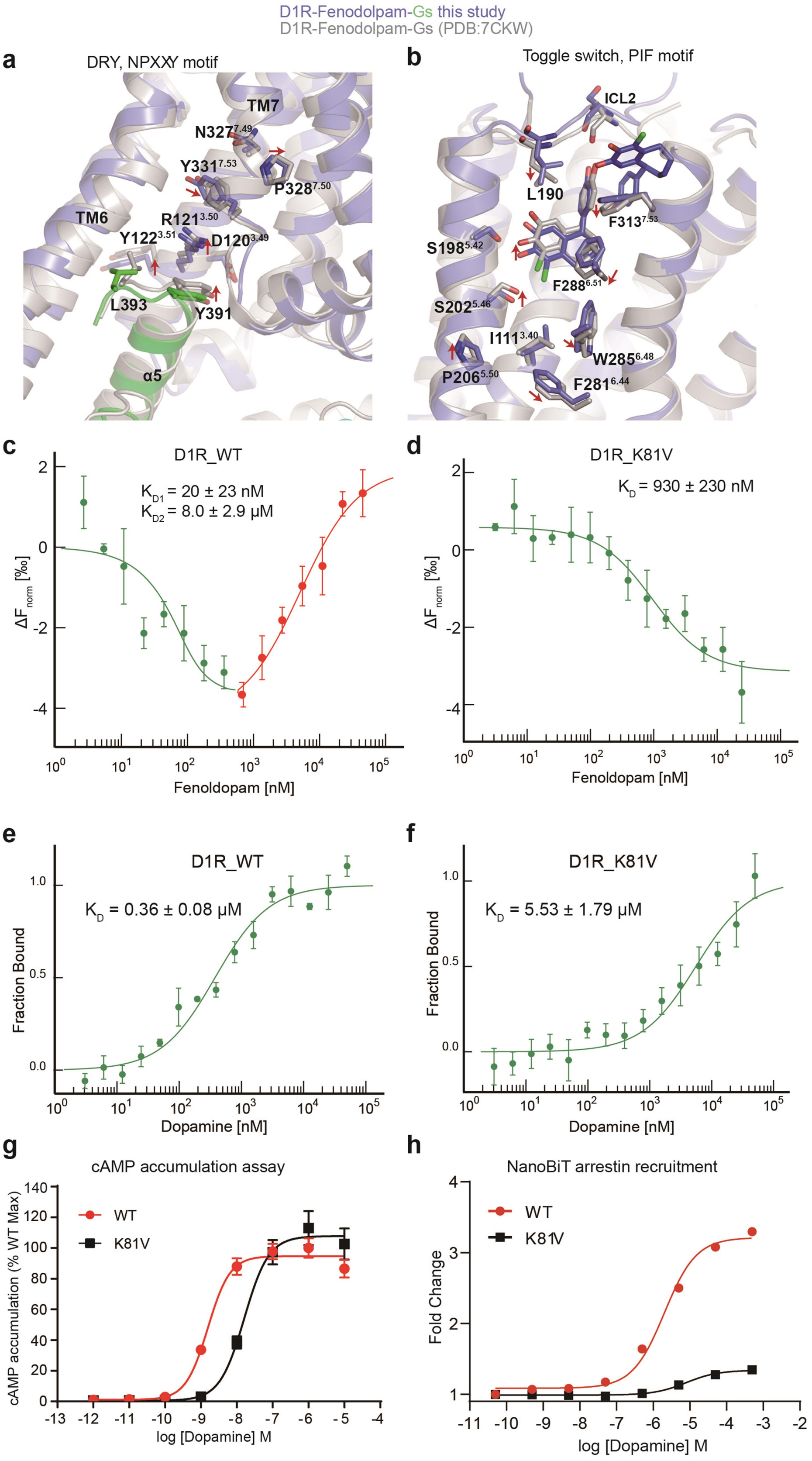

D1R-Fenodolpam-Gs this study
D1R-Fenodolpam-Gs (PDB:7CKW)
a
DRY, NPXXY motif
TM7
N327
P328
Y331
TM6
R121
Y122
D120
L393
Y391
α5
b
Toggle switch, PIF motif
ICL2
L190
F313
S198
F288
S202
I111
W285
P206
F281
c
D1R_WT
K_D1 = 20 ± 23 nM
K_D2 = 8.0 ± 2.9 μM
ΔF_norm [‰]
Fenoldopam [nM]
d
D1R_K81V
K_D = 930 ± 230 nM
ΔF_norm [‰]
Fenoldopam [nM]
e
D1R_WT
K_D = 0.36 ± 0.08 μM
Fraction Bound
Dopamine [nM]
f
D1R_K81V
K_D = 5.53 ± 1.79 μM
Fraction Bound
Dopamine [nM]
g
cAMP accumulation assay
cAMP accumulation (% WT Max)
WT
K81V
log [Dopamine] M
h
NanoBiT arrestin recruitment
Fold Change
WT
K81V
log [Dopamine] M

Supplementary Fig. 3. Structural comparison of the fenoldopam-bound D1R complex in this study with that in the previous study. a, Conformation changes of $D^{3.49}R^{3.50}Y^{3.51}$ and $N^{7.49}P^{7.50}xxY^{7.53}$ motif. **b,** Conformational changes of ligand binding pocket and the $P^{5.50}I^{3.40}F^{6.44}$ motif. **c,** MST binding experiment in which fenoldopam was titrated to the fluorescence-labeled D1R-mini-Gαs WT. The MST curve indicates a biphasic event. The high affinity (green) and low affinity phase (red) are independently fit to yield two dissociation constants, K_{D1} and K_{D2}. **d,** MST binding curve of the D1R-mini-Gαs K81V mutant titrated with fenoldopam reveals a single binding event. **e-f,** MST binding curve of D1R-mini-Gαs WT (**e**) and K81V mutant (**f**) titrated with dopamine. All MST binding curves are repeated in three independent times. Each data point are presented as mean values +/- SD. **g-h**, Concentration-response curve for D1R WT and the K81V mutant activated by dopamine measured using the cAMP accumulation assay (**g**) and NanoBiT β-arrestin recruitment assay (**h**). All data represent mean ± S.E.M from three independent experiments. The cAMP accumulation assay was performed in Expi293F cells transiently expressing the cAMP GloSensor. Response in the cAMP assay is expressed as percentage of maximal response of WT. Source data are provided as a Source Date file.

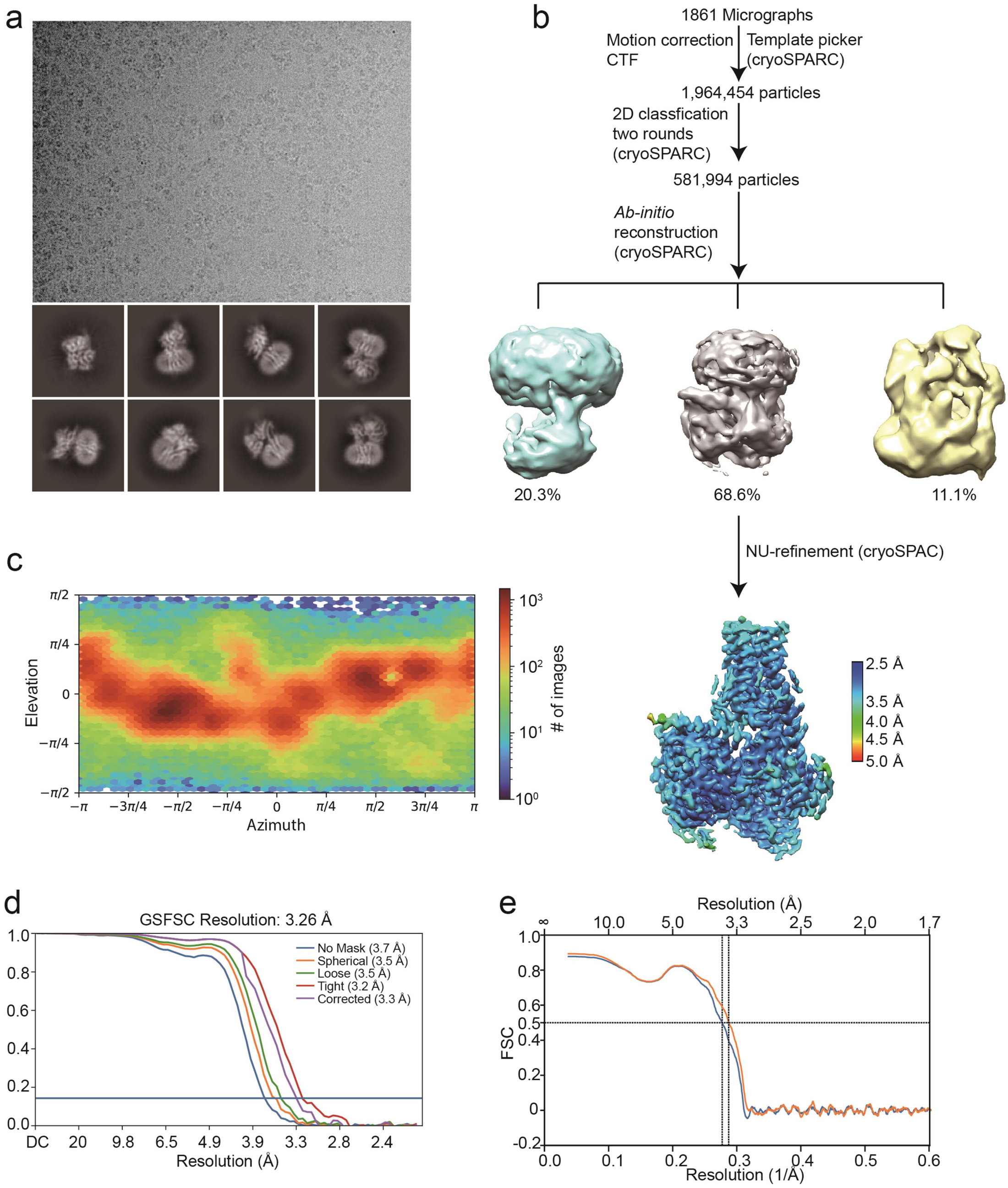

Supplementary Fig. 4. Cryo-EM data processing for the tavapadon-bound D1R complex. a, Representative cryo-EM image (top) and 2D class average (bottom). **b,** Cryo-EM workflow chart. **c,** Angular distribution plot. **d,** Gold standard FSC curves. **e,** FSC curves of model-to-map.

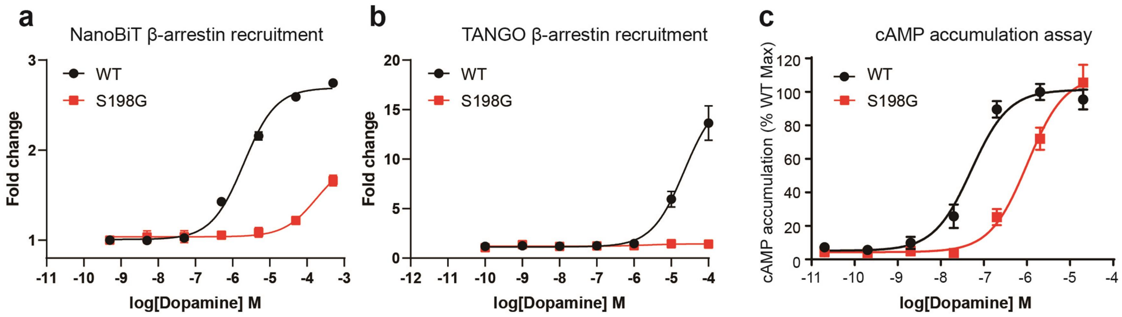

d

	NanoBiT β-arrestin recruitment assay				cAMP accumulation assay			
	Tavapadon		Dopamine		Tavapadon		Dopamine	
	pEC_{50} ±S.E.M.	E_{max}	pEC_{50} ±S.E.M.	E_{max}	pEC_{50} ±S.E.M.	E_{max}	pEC_{50} ±S.E.M.	E_{max}
WT	6.77±0.075	1.574	5.69±0.019	2.727	8.38±0.127	98.09	7.28±0.111	101.3
S198G	7.61±0.032	2.387	3.57±0.026	2.049	8.30±0.268	44.68	5.99±0.051	109.7
WT Biased factor (G protein versus Arrestin)	0.24							
S198G Biased factor (G protein versus Arrestin)	-2.20							

Supplementary Fig. 5. Tavapadon is a more potent β-arrestin-biased agonist for D1R S188G compared to WT. a-c, Concentration response curves for D1R WT and S198G mutant using NanoBiT (**a**) and TANGO (**b**) β-arrestin recruitment assays, and cAMP accumulation assay (**c**). All data represent mean ± S.E.M from three independent experiments. **d**, Summary of the maximum response (E_{max}) and pEC_{50} from the NanoBiT β-arrestin recruitment assay and the cAMP accumulation assay for tavapadon and dopamine. Bias factors for tavapadon were calculated using dopamine as a reference ligand. Source data are provided as a Source Date file.

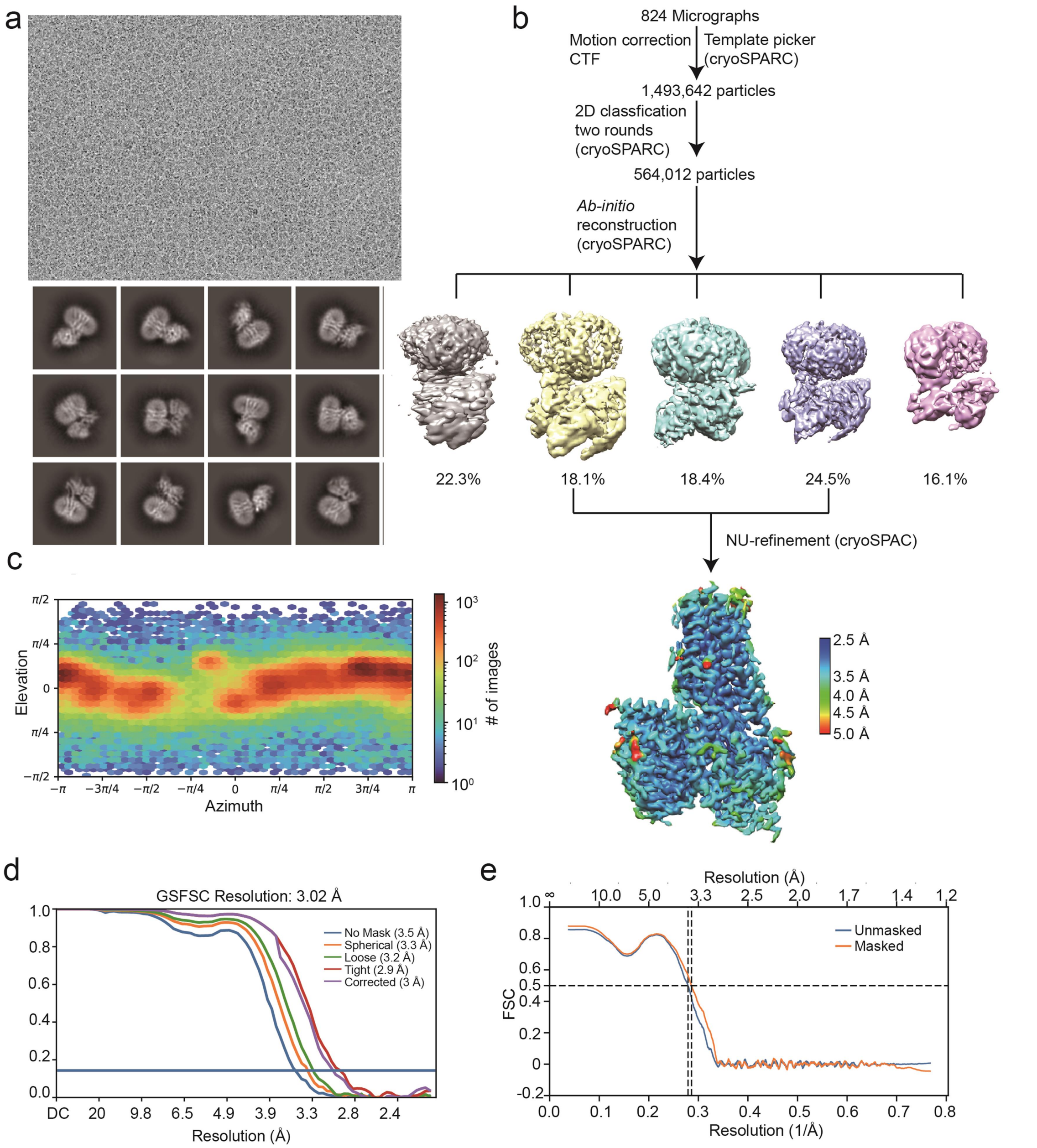

Supplementary Fig. 6. Cryo-EM data processing for the D1R complex simultaneously bound to dopamine and LY3154207. a, Representative cryo-EM image (top) and 2D class average (bottom). **b,** Cryo-EM workflow chart. **c,** Angular distribution plot. **d,** Gold standard FSC curves. **e,** FSC curves of model-to-map.

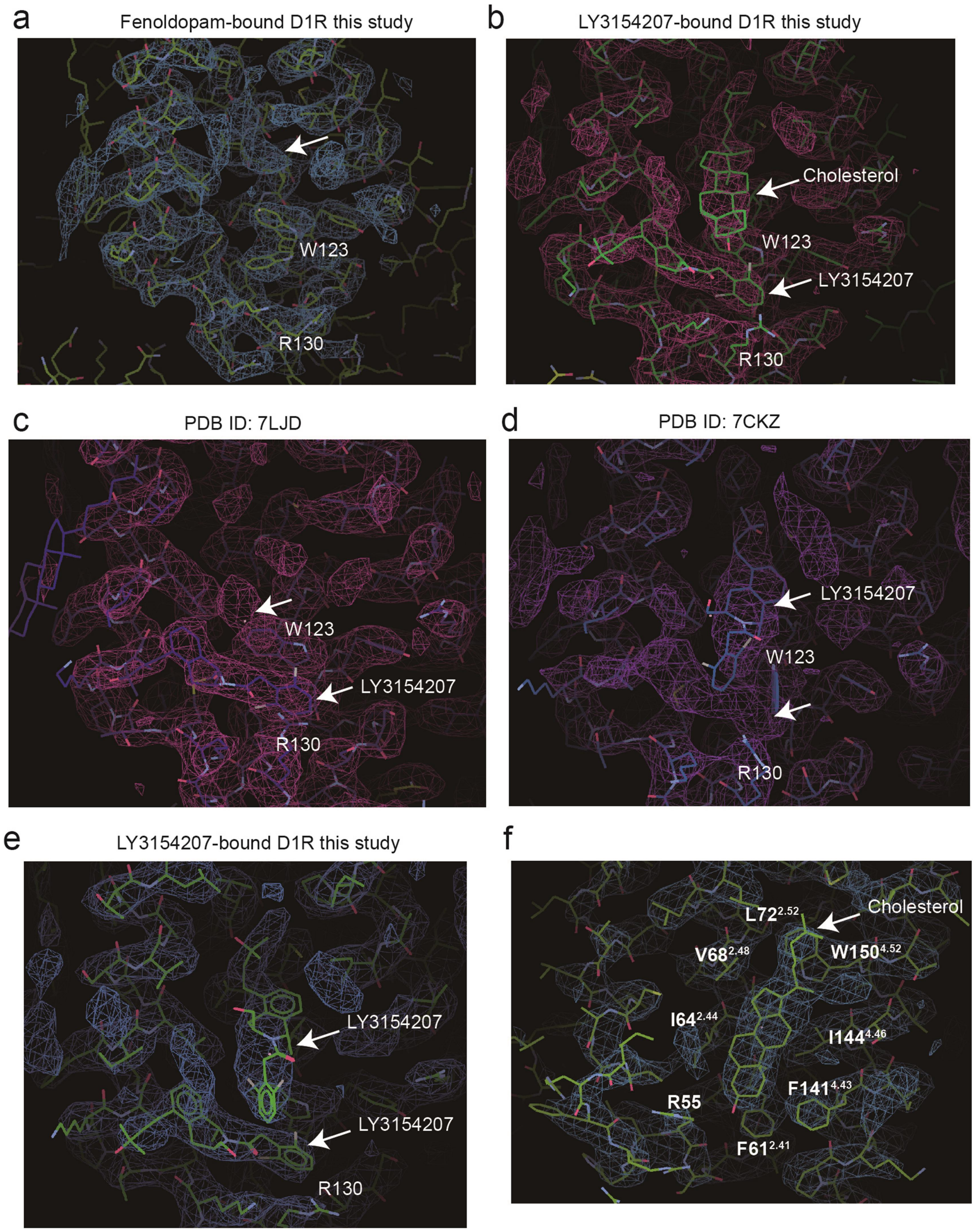

Supplementary Fig. 7. Representative EM density map. a, EM density for the region of the fenoldopam-bound D1R complex corresponding to the LY3154207 binding site in the structure of the LY3154207-bound D1R complex from this study. The arrow indicates the partially resolved density of cholesterol. **b**, Cholesterol and LY3154207 are modeled in EM density map from this study. **c-d,** LY3154207 and corresponding EM density map from the previously published structures of the LY3154207-bound complex (PDB ID: 7LJD and 7CKZ). **e,** Two LY3154207

molecules are modeled in EM density map from this study. **f,** EM density for the cholesterol binding site among TM2, TM3 and TM5 from this study.

Supplementary Table 1. Cryo-EM data collection and refinement statistics.

EM data collection statistics

Protein	D1R-G-Nb35 -Fenoldopam	D1R-G-Nb35 -Tavapadon	D1R-G-Nb35 -LY3154207
EMDB			
Microscope	FEI Titan Krios	FEI Titan Krios	FEI Titan Krios
Voltage (kV)	300	300	300
Detector	Gatan K3	Gatan K3	Gatan K3
Magnification (nominal)	64000	64000	64000
Pixel size (Å/pix)	1.087	1.087	1.087
Flux (e^-/pix/sec)	22	22	22
Frames per exposure	32	32	32
Exposure (e^-/Å^2)	50	50	50
Defocus range (μm)	0.8-2.1	0.7-2.0	0.6-2.2
Micrographs collected	1035	1861	824
Particles extracted/final	124,932	399,390	234,629
Map sharpening B-factor	-161.4	-195.8	-153.2
Unmasked resolution at 0.143 FSC (Å)	3.6	3.7	3.5
masked resolution at 0.143 FSC (Å)	3.2	3.3	3.0

Model refinement and statistics

	D1R-G-Nb35 -Fenodolpam	D1R-G-Nb35 -Tavapadon	D1R-G-Nb35 -LY3154207
PDB			
Composition			
Amino acids	1036	1032	1036
Ligand	3	2	4
RMSD bonds (Å)	0.004	0.004	0.004
RMSD angles (º)	0.707	0.797	0.776
Mean B-factors			
Amino acids	104.51	60.94	101.16
ligand	65.18	56.11	78.93
Ramachandran			
Favored (%)	97.84	97.24	96.76
Allowed (%)	2.16	2.76	3.24
Outliers (%)	0	0	0
Rotamer Outliers (%)	0.57	0.34	0
Clash score	13.18	13.94	16.18
C-beta outliers (%)	0	0	0
CC (mask)	0.73	0.79	0.73
MolProbity score	1.67	1.79	1.91
EMRinger score	1.80	2.37	2.55
